# THE EFFICACY OF MUSIC AND DEMENTIA EDUCATION PROJECT ON HEALTH CARE PROFESSIONAL STUDENTS: PRELIMINARY OUTCOMES

**DOI:** 10.1093/geroni/igac059.2789

**Published:** 2022-12-20

**Authors:** Jiaming Shi, Fei Sun, Jen Hirsch, Dan Cohen, Ha-Neul Kim

**Affiliations:** Michigan State University, East Lansing, Michigan, United States; Michigan State University, East Lansing, Michigan, United States; Michigan State University, East Lansing, Michigan, United States; Right to Music, New York City, New York, United States; Michigan State University, East Lansing, Michigan, United States

## Abstract

This study aims to improve dementia literacy and competence to use music in practice with people with dementia among students in health professional fields. The eight-week curriculum was developed by a gerontology social worker and a subject matter expert on personalized music use for people with dementia. Two focus groups were completed in Spring of 2022 with people living with dementia and care partners to refine the curriculum and include information that those with lived experience felt was important for future health care professionals to know. The study used 2 single group designed cohorts of students who received the 8-week educational intervention over Zoom. Each cohort included 2 persons living with dementia (PWD) as co-trainers. Pre- and post- surveys included a modified Alzheimer’s Disease Knowledge Scale and Dementia Attitude Scale. Participants (n=17) included Social Work = 8, Nursing = 2, Psychology =3, and Others =4. Total of 11 student participants completed both pre-test and post-test surveys. Paired t-tests were used to compare pre-post differences. At post-test, participants reported increased dementia knowledge (M=2.45, p=.0027); increased dementia attitudes (M=13.18, p=.0057); and improved competency on using music with PWD (M=.97, p=.0045)compared to the baseline scores. The 8-week intervention showed a statistically positive impact on dementia attitudes and competency of music use. The findings of this study laid the groundwork for future studies on training current service professionals and comparing their practice after the training to non-trained professionals to measure the impact of the training in intervention.

